# COVID-19 Diagnostic and Management Protocol for Pediatric Patients

**DOI:** 10.6061/clinics/2020/e1894

**Published:** 2020-04-13

**Authors:** Ana Paula de Carvalho Panzeri Carlotti, Werther Brunow de Carvalho, Cíntia Johnston, Isadora Souza Rodriguez, Artur Figueiredo Delgado

**Affiliations:** I Departamento de Pediatria, Hospital das Clinicas, Faculdade de Medicina de Ribeirao Preto, Universidade de Sao Paulo, Ribeirao Preto, SP, BR; II Instituto da Crianca e do Adolescente (Icr), Hospital das Clinicas HCFMUSP, Faculdade de Medicina, Universidade de Sao Paulo, Sao Paulo, SP, BR

**Keywords:** COVID-19, Pediatric Critical Care Medicine, Infection, Ventilatory Support, Diagnostic Criteria

## Abstract

This review aims to verify the main epidemiologic, clinical, laboratory-related, and therapeutic aspects of coronavirus disease 2019 (COVID-19) in critically ill pediatric patients.

An extensive review of the medical literature on COVID-19 was performed, mainly focusing on the critical care of pediatric patients, considering expert opinions and recent reports related to this new disease. Experts from a large Brazilian public university analyzed all recently published material to produce a report aiming to standardize the care of critically ill children and adolescents.

The report emphasizes on the clinical presentations of the disease and ventilatory support in pediatric patients with COVID-19. It establishes a flowchart to guide health practitioners on triaging critical cases.

COVID-19 is essentially an unknown clinical condition for the majority of pediatric intensive care professionals. Guidelines developed by experts can help all practitioners standardize their attitudes and improve the treatment of COVID-19.

## INTRODUCTION

Coronavirus disease 2019 (COVID-19) is a viral respiratory illness caused by severe acute respiratory syndrome coronavirus 2 (SARS-CoV-2), a single-stranded RNA virus that most likely originated in bats. The virus is thought to spread mainly from person-to-person via close contact (the virus can be transferred from the hands to the eyes, nose, or mouth) and respiratory droplets (produced when an infected person coughs or sneezes). There is no evidence of vertical transmission or transmission via breastfeeding. Transmission from asymptomatic or mildly symptomatic carriers or during the incubation period, estimated to be between 1 and 14 days (mean, 5 days), can also occur; 95% of patients develop symptoms up to 12.5 days after exposure. This gave rise to the established quarantine period of 14 days after exposure 1,2.

### Clinical presentation

**Figure 1:**
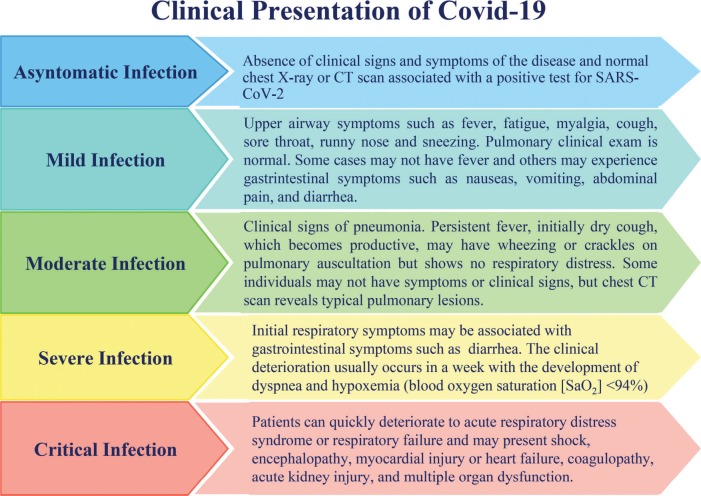
Clinical Presentation of COVID-19.

The clinical spectrum of COVID-19 ranges from being asymptomatic to being in severe acute respiratory distress (Figure 1) 3.

According to a case series of 2143 pediatric patients 3 registered in the China Center for Disease Control and Prevention (CDC) database, 731 cases were confirmed using laboratory testing, 94 (4.4%) patients were asymptomatic, 1091 (50.9%) patients presented with mild symptoms, and 831 (38.8%) patients presented with moderate symptoms. Only 125 (5.8%) patients developed severe or critical disease. Younger children were more susceptible to severe or critical symptoms (10.6% <1 year old *vs.* 3% ≥16 years old); there were 13 critical cases, and in seven (53.8%) of them the patient was less than 1 year old. Only one death was reported, that is, of a 14-year-old boy.

According to another series of 171 pediatric cases 4 (1 day to 15 years old; median, 6.7 years) admitted to a hospital in Wuhan, China, all patients tested positive for COVID-19, 27 (15.8%) were asymptomatic, 33 (19.3%) had upper airway symptoms, and 111 (64.9%) had pneumonia. Seventy-one pediatric patients presented with fever (41.5%) which lasted 1 to 16 days (median, 3 days). Three patients were admitted to the intensive care unit; all of them had comorbidities such as hydronephrosis, leukemia (during chemotherapy), and intussusception. The patient presenting with intussusception was 10 months old; the patient's condition deteriorated, leading to multiple organ dysfunction and death.

Various cutaneous rashes have been recently observed in some pediatric cases with variable clinical presentations 5,6.

### Laboratory findings

The white blood cell count can be normal or reduced. A series of 171 pediatric cases of COVID-19 reported leukopenia in 26.3% of patients; only 3.5% developed lymphocytopenia. C-reactive protein levels can be normal or elevated. Severe or critical cases may be accompanied by an elevation in hepatic and muscular enzyme levels and high D-dimer levels 2,4.

### Imaging findings

During the initial phase of illness, chest radiography findings can show signs of pneumonia, such as small irregular lung opacities and interstitial alterations, usually affecting peripheral areas. Ground glass opacities (GGO) and consolidation may be observed in severe cases. Pleural effusion is uncommon. Chest computed tomography also exhibits GGO and segmental consolidation in both lungs. Children presenting with severe infection may show lobar consolidation bilaterally 2. Lung ultrasonography (US) exhibits single or grouped, usually bilateral, pneumogenic-type vertical artifacts and/or small areas of white lung. Advanced COVID-19 pneumonia is characterized by evident consolidation, particularly in the posterobasal regions, and widespread patched artifactual changes, similar to those in acute respiratory distress syndrome (ARDS) 7. Thoracic electrical impedance tomography can be used to monitor the distribution of regional ventilation in patients with ARDS and to identify refractory hypoxemia that requires alveolar recruitment maneuvers.

### Detection of etiological agent

The detection of SARS-CoV-2 nucleic acid using real-time reverse transcriptase-polymerase chain reaction (RT-PCR) is the reference standard for COVID-19 diagnosis. The virus can be detected in upper airway or inferior airway secretions (nasopharynx swab or tracheal aspirates [if intubated], sputum, and bronchoalveolar lavage), blood, urine, and stool 1,8.

In a case series of 10 pediatric patients from Shanghai 9, viral RNA was detected in the nasopharynx of all patients from 4 to 48h after the beginning of symptoms up to 6 to 22 days (mean, 12 days) after the first day of illness. In five patients, SARS-CoV-2 RNA was detected in the stool until 18 to 30 days after the initial symptoms.

According to the 22-38 SS Resolution, 3-17-2020, from São Paulo's State Health Secretary 10, RT-PCR for SARS-CoV-2 should be performed only for patients in a severe or critical condition, those in sentinel units, and health care professionals with symptoms typical of COVID-19. The test should not be performed in asymptomatic individuals.

### Diagnostic criteria

**Figure 2:**
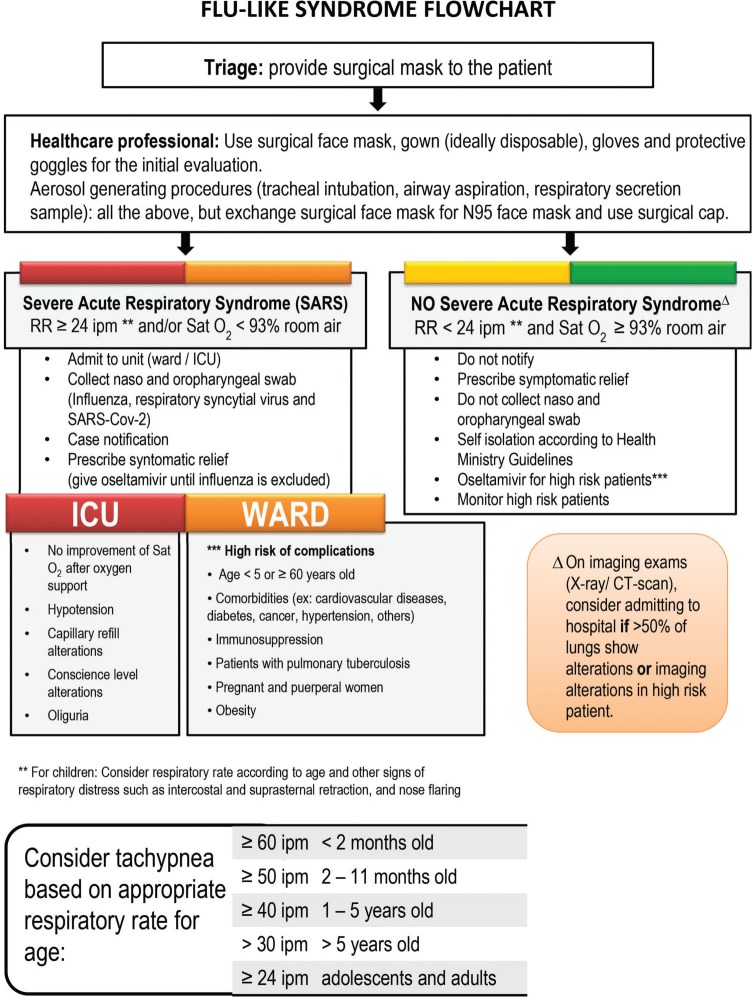
Flu-like syndrome flowchart.

A diagnosis is made considering clinical findings, epidemiology, and laboratory testing to confirm SARS-CoV-2 infection 2. Figure 2 shows the flowchart for patients with flu-like symptoms as proposed by the São Paulo State Health Secretary 10,11.

### Differential diagnosis

#### Other viral respiratory illnesses

Respiratory syncytial virus, influenza, parainfluenza, adenovirus, and metapneumovirus are frequent causes of lower airway infection in children and have similar clinical presentation to COVID-19. The diagnosis can be made by identifying the etiological agent in respiratory secretions using polymerase chain reaction.

#### Bacterial pneumonia

The main clinical manifestations of bacterial pneumonia are high fever and toxemic state. Frequently, there is leukocytosis with neutrophilia and increased numbers of younger cells. Blood and aspirate tracheal cultures are very important. Bronchoalveolar lavage (BAL) in intubated patients can be very useful in making a diagnosis.

#### Atypical pneumonia

*Mycoplasma pneumoniae* and *Chlamydia pneumoniae* are important causative agents of community-acquired pneumonia in children, and serologic tests can be used to confirm the diagnosis 2.

### Therapeutic management

The four main principles for adequate therapeutic management are early identification, early isolation, early diagnosis, and early treatment. When dealing with a case of suspected COVID-19, the patient should be kept in a single room with all precautions to prevent and control infections before laboratorial confirmation. Mild cases should be treated with symptomatic relief medication, preferably paracetamol or dipyrone, to control fever. Antiviral agents, including oseltamivir, ribavirin, ganciclovir, remdesivir, lopinavir, and ritonavir, have been used to reduce the viral load to prevent potential respiratory complications but with no apparent benefits thus far 1,2,12. A Chinese study 13 of more than 100 patients reported that chloroquine was secure and effective in treating COVID-19-associated pneumonia, inhibiting exacerbation of pneumonia, reducing radiographic alterations, promoting virus elimination, and decreasing the period of time of the disease. Another French study 14 enrolled 20 patients with COVID-19 and showed that treatment with hydroxychloroquine was associated with cure of the viral infection, with the utilization of azithromycin leading to increased benefit. There are other current studies analyzing the drug's efficiency in critically ill patients with a higher risk of death and considering the use of hydroxychloroquine/chloroquine (5–10 mg/kg/day of basic chloroquine for 10 days) and azithromycin (10 mg/kg on the first day, followed by 5 mg/kg/day for 4 days with a maximum dose of 30 mg/kg or 1,500 mg) 14-16.

Severe cases with respiratory distress and/or hypoxia (SaO_2_ <94%) (Severe Acute Respiratory Syndrome) should be admitted to the hospital. Indications for ICU admission are: respiratory failure requiring mechanical ventilation, shock or other organ dysfunction requiring treatment 9,17. The main characteristic of the critical cases is the occurrence of ARDS with hypoxemic acute respiratory distress and bilateral pulmonary infiltrates that are not explained by cardiac dysfunction or fluid overload. The main treatment principles of ARDS are as follows 17-20:

- Early tracheal intubation – rapid sequence intubation is the best practice in this situation. Preoxygenation should be performed using a flexible nasal cannula (up to flows of 4 L/min) or a reservoir mask with a lower flux to maintain an SaO_2_ >93%. Positive pressure ventilation with a bag-valve-mask or other similar apparatus should be avoided to not generate aerosols. Sedation can be performed using fentanyl (1–2 µg/kg) or ketamine (1–2 mg/kg, if there is no contraindication such as pulmonary hypertension) and neuromuscular blocking with rocuronium (0.6–1.2 mg/kg), preferably. Video laryngoscopy should be utilized if available.
- Non-invasive ventilation (NIV) should be avoided because of the high risk of aerosol dispersion and contamination among health practitioners.
- Protective mechanical ventilation using a pressure controlled or volume cycled, with a low tidal volume (around 6 mL/kg) and plateau pressure ≤30 cmH_2_O. The positive end-expiratory pressure (PEEP) should be titrated such that FiO_2_ is reduced (≤50%), with PaO_2_ >60 mmHg and SaO_2_ >90–93%. The general recommendation is to start with PEEP at 5–6 cmH_2_O and increase progressively to 12–14 cmH_2_O if necessary. It is very important to maintain a driving pressure (plateau pressure – PEEP) <15 cmH_2_O and tolerate hypercapnia with pH >7.2 (permissive hypercapnia), except for patients with pulmonary hypertension.
- Prone positioning, especially if PaO_2_/FiO_2_ <150 mmHg. The patient should be maintained in this position at least 18 hours per day with oximetry and capnography monitoring.
- Closed tracheal aspiration systems should be used.
- Treatment with nitric oxide and/or sildenafil (0.5–2 mg/kg/dose each 4–6 hours with a maximum of 20 mg/dose each 8 hours) for patients with persistent hypoxemia. Veno-venous extracorporeal membrane oxygenation (ECMO) can be used in patients with PaO_2_/FiO_2_ <70–80 mmHg in whom conventional treatment (protective mechanical ventilation, prone positioning, nitric oxide/sildenafil) was not successful, according to its availability in the hospital.
- Conservative fluid therapy, including volume restriction in patients with hemodynamic stability, starting with 50% of the normal recommendation based on the Holliday-Segar rule, and adjustments according to the fluid balance and hemodynamic clinical conditions.

Patients with wheezing and lower airway obstruction should be treated with the aim of reducing respiratory droplet dispersion inside the environment. Aerosol bronchodilators should be avoided and spray or dosimetric inhalers should be used. It is possible to utilize NIV in some cases but it should use a mechanical ventilator with a double circuit, with filters in both parts (to warm and humidify in the inspiratory circuit and a high-efficiency particle separator in the expiratory circuit). It is necessary to emphasize that NIV support has been used in isolation hospital rooms with negative pressure only. After 1 to 2 hours of non-invasive mechanical ventilation without adequate evolution, tracheal intubation is recommended. Mechanical ventilation support should avoid pulmonary hyperinsufflation, auto-PEEP, and barotrauma. Pressure-controlled ventilation is preferred in synchronized intermittent mandatory ventilation with support pressure, low breathing rate, inspiration/expiration time rate of 1:3–1:4 and PEEP=5 cmH_2_O.

Antibiotics should be used only in patients with secondary bacterial infections on the basis of the culture and antibiogram results. Corticosteroids can suppress lung inflammation but also inhibit immune responses and pathogen clearance, and should be avoided except for those with a specific indication (*e.g.*, bronchospasm or some cases of septic shock) 1,2. There is a report that intravenous immunoglobulin treatment in a 3-year-old critically ill child was effective 21. Patients with cardiocirculatory dysfunction and shock should be treated with fluid therapy or vasoactive/inotropic drugs. Milrinone (0.1–1.0 µg/kg/min) or dobutamine (5–15 µg/kg/min) can be useful in patients with a low cardiac index as a consequence of pulmonary hypertension and normal arterial pressure. Epinephrine at an inotropic dose (≤0.3 µg/kg/min) can be used in patients with hypotension. In children and adolescents with other organic dysfunctions, continuous renal replacement therapy may be necessary because renal dysfunction is an indicator of a poor prognosis 1,2,7. Microthrombosis and associated ischemic events are very common (*e.g.*, strokes), mainly in adolescents and adults. D-dimer levels should be monitored frequently.

Appropriate medical transport for the patient (and their parent/caregiver) to the hospital should be provided by medical transport teams. Community pediatricians should advise parents to wait for this service and to not seek secondary care themselves, for example, by going to the hospital via car or public transport 22.

## AUTHOR CONTRIBUTIONS

Carlotti APCP, Carvalho WB, Johnston C, Rodriguez IS and Delgado AF were responsible for the study conceptualization, supervision and manuscript original drafting, editing and review.
